# Trisomy 21 Alters Motor Coordination, Vocal Communication, and Cerebellar Circuit Connectivity in the TcMAC21 Mouse

**DOI:** 10.21203/rs.3.rs-5724831/v1

**Published:** 2025-09-09

**Authors:** kuangfu Hsiao, Rachel Stander, Nithilah Ayyappan, Meike van der Heijden

**Affiliations:** Children’s National Hospital

## Abstract

Individuals with Down syndrome (DS) frequently face challenges with motor control and coordination, affecting their daily physical movements. Speech and language difficulties are also well-documented in DS, but the degree to which these challenges relate to underlying motor coordination deficits remains poorly understood. Using a DS mouse model containing triplication of a nearly complete human chromosome 21, the TcMAC21 mouse, we identified cerebellar circuit dysfunction as a convergent mechanism for both motor and linguistic impairments. Systematic analysis revealed disrupted Purkinje cell organization throughout development, accompanied by specific deficits in cerebellar-dependent behaviors including motor learning, vocalization, and maternal care. Structural measurements and targeting by cell-specific DREADDs uncovered disrupted calcium homeostasis in Purkinje neurons during critical periods of climbing fiber refinement as one contributing factor. In vivo neurophysiological recording in TcMAC21 mice revealed reduced cerebello-thalamic synchrony during locomotor activity. These findings identify calcium signaling as a key developmental pathway linking chromosomal trisomy to cerebellar circuit dysfunction, providing a novel framework for understanding both motor and linguistic deficits in DS that extends beyond traditional cortico-centric models.

## 1. INTRODUCTION

Down syndrome (DS) results from an extra copy of human chromosome 21 (HSA21) and, in addition to causing intellectual disability (ranging from mild to moderate), significantly impacts motor skills and speech, thereby significantly affecting independent living and communication [1,2]. Cerebellar hypoplasia is consistently observed in both DS patients and mouse models [3,4]. The convergence of these prominent symptoms in DS, along with growing evidence implicating cerebellar dysfunction in these deficits [5–8], as the cerebellum regulates motor coordination, language, and social behaviors, together suggests cerebellar dysfunction may underlie motor and speech deficits in DS.

Beyond granule cell loss and a gross reduction in cerebellar size, HSA21 gene amplification disrupts cerebellar physiology by dysregulating calcium homeostasis. HSA21 genes like *PCP4*, *RCAN1*, and *DYRK1A* suppress the calcineurin pathway, compromising Purkinje neuron calcium buffering and synaptic function [9–11]. This disruption affects both parallel fiber and climbing fiber inputs, potentially underlying cerebellar learning deficits [12]. The olivocerebellar circuit, particularly climbing fiber inputs to Purkinje neurons, plays a crucial role in motor learning and coordination. Climbing fibers provide instructive signals that shape cerebellar output through their precise topographical organization in parasagittal zones [13,14]. These climbing fiber inputs drive complex spikes in Purkinje neurons, modulating synaptic plasticity and motor adaptation through error prediction and motor correction [15,16]. Climbing fiber inputs shape Purkinje cell activity, which in turn modulates cerebellar nuclear output to motor thalamus, forming a precisely coordinated circuit essential for motor learning and execution [17,18].

While previous DS mouse models have provided valuable insights, they have significant limitations in genetic representation and phenotypic stability [19,20]. The introduction of a more advanced model, the TcMAC21 mouse, which contains 93% of the protein-coding genes on HSA21 making it the most complete genetic model to date, offers a more accurate representation of HSA21 [21,22]. This model circumvents many of the imperfections found in Ts65Dn and provides a new avenue for studying cerebellar function in DS. However, its cerebellar phenotypes, particularly in movement control and vocalization, remain unexplored. Here, we investigate how trisomy affects cerebellar circuit development and function using this model, focusing on the relationship between calcium homeostasis disruption and circuit-level dysfunction in motor and communication deficits.

## 2. MATERIALS AND METHODS

All experimental protocols were approved by the Institutional Animal Care and Use Committee (IACUC) at Children’s National Medical Center.

### Mice

TcMAC21 mice (JAX #035561) and euploid littermate controls were maintained by breeding female TcMAC21 to male B6D2F1/J (JAX #100006) mice. *Pcp2*-Cre mice (JAX #004146) were maintained on C57BL/6 background. Breeder mice (6–8 weeks) were obtained from Jackson Laboratory. Experiments used animals from at least two litters per group. Mice were housed under 12:12 light/dark cycle at 22±2°C with ad libitum food and water.

### Animal Behaviors

#### ErasmusLadder Analysis

Motor control performance test to measure cerebellar function was assessed by daily testing of the ErasmusLadder task according to the procedure of Vinueza Veloz et al.[23], and was modified with test parameters as previously described [24]. Open field testing was performed as previously described [25].

#### Pup Separation Vocalization

Pup separation vocalizations were recorded by isolating pups (P8) individually in a soundproof chamber for 5 minutes as previously described [26]. Vocalizations were categorized by USVs (25–130 kHz). We quantified vocalization characteristics (mean frequency, range, duration, and tonality) using DeepSqueak’s automated calculations based on user-identified calls [27].

#### Pup Retrieval Test

Postpartum dams were given 24h for nest building. On P3 and P5, three pups were placed in different corners of the home cage (30×45×15 cm) with dam in the fourth corner. Tests occurred in dark phase under red light as previously described [26]. Retrieval latency was recorded over 10 min, with 600s maximum score if unsuccessful.

### Viral Constructs

The Cre-inducible adeno-associated virus (AAV) vectors expressing hM3D DREADD with ciliatargeting-sequence (CTS), hM3D-CTS, were generated by subcloning into AAV.CAG-FLEX vector under the control of loxP sitesThe cilia targeted hM3D-CTS DREADD and AAV.CAGFLEX were gifts from Drs. Gregory Pazour (University of Massachusetts Medical School) [28], Bryan Roth (University of North Carolina)[29], Chun-Li Zhang (RRID:Addgene_45560; RRID:Addgene_44361; RRID:Addgene_178583).

### Animal Surgical Procedures

Surgical procedures and viral injections were carried out under protocols approved by the Institutional Animal Care and Use Committee (IACUC) at Children’s National Medical Center.

#### Systematic Intra-Cisterna Magna Viral Delivery and Chemogenetic Manipulation

Single Intra-Cisterna Magna (ICM) injections were performed as previously described in *Pcp2Cre* mouse pups on postnatal day 2–3.. We systematic delivered conditional viral constructs (AAV.CAG-FLEX-hM3D-CTS) to P2 neonate via intra-cisterna magna (ICM) injection, which enhances cerebellar targeting while minimizing frontal cortex spread [30]. Briefly, *Pcp2-Cre* neonates were cryoanesthetized and subsequently placed on a cold metal plate. A 30-gauge needle was used to pierce the skull 2 mm posterior to lambda at the midline, and 4 μl of AAV (AAV.CAG-FLEX-hM3D-CTS or AAV.CAG-FLEX-TdTomato) was injected into each cisterna magna (1.0E10 GC). AAV.CAG-FLEX-TdTomato and Custom AAV.CAG-FLEX-hM3D-CTS

AAV production was carried out by Addgene and at Vigene Biosciences, respectively. Neonatal mice were kept with parent until weaned. Mice were sacrificed at set time points as follow: 7 weeks (n = 5 per group) and 2 months (n = 6 per group) post-injection. Of note, the former groups were being euthanized for biochemical and histological analysis without motor training. For Purkinje neuron calcium dysregulation during development, mice were given clozapine-Noxide (cno) dissolved in 0.9% saline at 1mg/kg or saline only, twice per day. Administration of cno took place every day P9–21 for preadolescent activation.

#### Stereotaxic Virus Injection and Cannula Implantation

In multi-fiber photometry experiments, we utilized a red fluorescent calcium sensor protein [31] to contrast the peri-centromeric GFP on the HSA21q-MAC [22] to independently record neural calcium activity by spectral separation. Stereotaxic surgeries were performed as previously described [25]. AAV vectors expressing hM3D-CTS DREADD were generated in AAV.CAG-

FLEX vector. For ICM injection, P2–3 Pcp2-Cre pups received 4μL AAV (1.0E10 GC) 2mm posterior to lambda. For photometry, AAV1-CAG.Flex.NES-jRCaMP1a was injected into cerebellar nuclei (AP:−6.13, ML:±1.40, DV: −3.60mm) and AAVrg.EF1a.Cre/AAV1Syn.NES.jRCaMP1a into ventrolateral thalamus (AP: −0.9, ML: ±1.00, DV: −3.75mm). Optical fibers (400μm, 0.48NA) were implanted above injection sites.

The expression of jRCaMP1 was confirmed as shown (Supplementary Figure 3A and 3B).

### Histology & Immunohistochemistry

Tissue processing and immunocytochemistry were performed exactly as described[32]. Mice were perfused with ice-cold PBS followed by 4% PFA. Brains were post-fixed for 24h at 4°C, cryoprotected in 30% sucrose, and sectioned at 40μm. Antibodies used: Calbindin D-28K (CB300, Swant), anti-VGluT2 (#135418, SYSY), anti-HSP25 (ADI-SPA-801-F), anti-VGAT (#131004, SYSY). Sections were imaged using Nikon Ti2 confocal microscope with 10×/0.45NA (lobe size), 20×/0.75NA (Hsp25 pattern), or 63×/1.40NA objectives (synaptic markers). Z-stacks were analyzed using IMARIS software (OXFORD Instrument).

### Purkinje neuron sagittal stripe gene expression quantifications

Cerebella from MAC21 mice and littermates (n=3 for P7/P14, n=4 for adult) were analyzed for Hsp25 distribution. Sections (40μm, 200μm intervals) were evaluated within 2mm square regions of lobular IX/X. Coexpression of Hsp25+/Calb+ and Hsp25−/Calb+ cells was quantified using ImageJ (Rasband, W.S., ImageJ, NIH, MD).

### *In Vivo* two-region Photometry Recordings

#### Photometry Setup

Excitation of the 560 nm (imaging) and 405 nm (isosbestic control) wavelengths were provided by commercially available photometry system (Neurophotometrics, Model FP3002) which are controlled via the open-source software Bonsai [33]. Excitation light is directed on to a custom branching fiberoptic patchcord of three bundled 400 μm diameter 0.22NA fibers (BFP(3)_400/440/900–0.22_2m_FCM*−3×FC, Doric Lenses) by objective lens

(Neurophotometrics, Model FP3002). RCaMP1a fluorescence from neurons below the fiber tip in the brain was transmitted via fiber optic patch cable back to the objective and were recorded. The multiple branch ends of the branching fiberoptic patchcord were connected to an array of fiberoptic rotary joints (FRJ_1×1_FC-FC, Doric Lenses) and coupled to two lowautofluorescence patchcords (MFP_400/430/1100–0.57_1m_FC-ZF1.25_LAF, Doric Lenses) which is used to collect emission fluorescence from 1.25mm diameter light weight ferrules (MFC_400/430–0.48_ZF1.25, Doric Lenses) using a mating sleeve (Doric SLEEVE_ZR_1.25). Bulk activity signals were collected using the PVCAM software, and data were further postprocessed and analyzed using custom MATLAB scripts.

#### Voluntary Wheel Running with Photometry Recordings

Mouse with photometry implants was head-fixed on running wheel (diameter: 12 cm, width: 5 cm) which was housed in dimly lighted, sound attenuated box. Wheel was fitted with sensor to record rotations via a computerized monitoring system. Following four ten-minute habituation sessions (2/day) to the head-fixed conditions, wheel-running activity was monitored continuously for four consecutive days. The following parameters were recorded: Running velocity (m/min), Running bout frequency (number of discrete running episodes/day). A running bout was defined as any wheel rotation lasting ≥ 3 seconds, with intervals of > 10 seconds of inactivity denoting separate bouts. All measurements were conducted under standard laboratory conditions. Mice performed voluntary Wheel Running task while we recorded bulk calcium signals from two regions, the cerebellar nuclei (CN) and motor thalamus (VL), simultaneously. We recorded at 30 Hz frequency with excitation alternating between 560 nm (calcium dependent fluorescence) and 405 nm (calcium independent fluorescence) excitation wavelengths, resulting in an effective frame rate of 15 Hz, sufficient for capturing jRCaMP1a fluorescence dynamics. Multi-Fiber Photometry Data Processing was performed as previously described [25].

#### QUANTIFICATION AND STATISTICAL ANALYSIS

##### Behavior Statistical Reporting

Sample sizes were based on literature precedent, with randomized group assignment and blinded investigators. Five experimental cohorts included: Cohort 1 (n=18 males, P45–60) for open field/ErasmusLadder; Cohort 2 (n=24 pups, P7–9) for USVs; Cohort 3 (n=10 dams, P90120) for retrieval; Cohort 4 (n=10 males, P56–72) for photometry; Cohort 5 (n=30) for chemogenetics, subdivided equally into hM3D-CTS+cno, hM3D-CTS+vehicle, and RFP+cno groups. All mice were behaviorally naïve. Data were analyzed using GraphPad Prism 10 with repeated measures ANOVA. Detailed statistics are reported in Table 2.

##### Multi-Fiber Photometry Data Analysis

Task phase activity was quantified as area under the curve (AUC) of z-scored dF/F responses using MATLAB trapz function. To facilitate comparison across mice, F/F responses were zscored and shifted above 0. For regional correlations, Pearson’s correlation coefficients were calculated between brain regions. To control for long photometry responses, timeseries were circularly permuted (15–25 frame offset) during running bouts. State discrimination was quantified using discrimination index (*DI*):

*DI* = |mean (*correlation_running*) – mean (*correlation_stationary*)|/(std(*correlation_running*) + std(*correlation_stationary*)); *correlation_running*: Pearson’s r between cerebellar nuclei and motor thalamus during running epochs; *correlation_stationary*: Pearson’s r during stationary epochs.

## 3. RESULTS

### Impaired motor control and altered vocalizations in TcMAC21 mice

**Figure 1:**
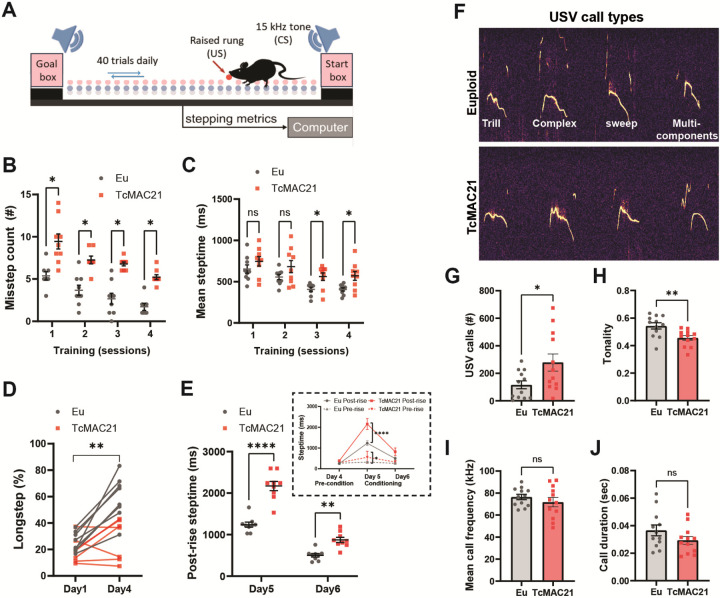
TcMAC21 mice exhibit lower motor control skill and altered vocalization. **(A)** Schematic presentation of the Erasmus Ladder which consists of a horizontal ladder situated between two shelter boxes. The mouse begins the task from inside the start box for a random time interval that varies between 9-s and 18-s before it is allowed to walk on the ladder. When the time interval has passed, the LED light in start box turns on and the mouse is allowed to start. The light remains on until the mouse reaches the goal box. Inter-limb coordination is tested during Day 1–4. Each daily session consisted of 40 trials, during which the mice had to walk back and forth between goal and start boxes. Mice usually stepped on the upper rungs and only infrequently touched the lower ones, referred to as missteps. Locomotion adaptation is tested during challenge sessions (Day 5–8) when the mice learned to adapt their walking patterns in response to a 15 kHz auditory stimulus (CS) preceding the appearance of a raised rung (US) in their pathway. The US was located on the right side of the mouse and specifically moved depended on the predicted position of the mouse on the ladder but was otherwise randomized. The blue and gray dots represent the upper and the lower rungs, respectively. The position of the obstacle is indicated with a red dot and arrow during the challenge sessions. **(B)** Plot of the mean of average number of missteps per trial during inter-limb coordination test. TcMAC21 mice made more missteps when transversing through rungs on a mouse-by-mouse basis. Eu: *n* = 9 mice, TcMAC21: *n* = 9 mice. **(C)** TcMAC21 steptime had a longer latency during inter-limb coordination test. **(D)** MAC21 mice made a lesser proportion adapting long-stride pattern. **(E)** The first day of the challenge session (Day 5), TcMAC21 exhibited a greater increase in mean latency (“post-rise step time”) in response to the raised rung. **(F)**Representative sonograms of the four types of neonatal separation vocalizations. **(G)** TcMAC21 neonates made significantly more total number of calls and **(H)** reduced vocalization tonality (signal-to-noise ratio) during separation-induced vocalizations. **(I)** No genotype difference in the mean vocalization frequency or **(J)** duration of calls produced. Data presented on a mouse-by-mouse basis (Eu: *n* = 12, TcMAC21: *n* = 12). Data represent mean ± S.E.M.; *p < 0.05; **p < 0.01; black: Eu, red: MAC21.

To determine whether humanized trisomic mice (TcMAC21) exhibit deficits in complex, multi-joint motor behaviors similar to patients with DS [1,34–36], we assessed locomotor deficits in TcMAC21 mice using the ErasmusLadder task [23,37,38], which evaluates inter-limb coordination and cerebellar learning (Figure 1A) while minimizing physical confounds [39]. Before motor testing, we evaluated signs of changes in limb development in young adults (femur length (mm): Eu 13.87±0.37, TcMAC21 Eu 13.57±0.65; tibia length (mm): Eu 18.07±0.42, TcMAC21 Eu 18.04±0.69; see Supplementary Figure 1A,B) and motivational or activity deficit (Open field maze, Supplementary Figure 1D-F). TcMAC21 mice could not be discriminated from control littermates in these assessments. This is particularly important because TcMAC21 mutants have shown less weight gain over time than euploid mice (Supplementary Figure 1C) [40]. During unperturbed training sessions, TcMAC21 mice exhibited motor impairments compared to euploid littermates, making more missteps (Figure 1B; 2way ANOVA repeated measures, trisomy effect F_(1, 12)_ = 30.64, *p*<0.001; session effect F_(3, 32)_ = 0.5022, *P*<0.001) and showing extended response times (Figure 1C; 2way ANOVA repeated measures, trisomy effect F_(1, 16)_ = 5.867, *p*=0.0277; session effect F_(2.319, 37.10)_ = 22.75, *p*<0.0001). By day 4, euploid mice adopted long-stride patterns to reduce steps between goals, while TcMAC21 mice did not (Figure 1D; 2way ANOVA repeated measures, trisomy effect F_(1, 13)_ = 12.05, *p*=0.0041, session effect F_(1, 13)_ = 30.01, *p*=0.0001).

In challenge sessions (days 4–8), mice encountered obstacle rungs (US) preceded by warning tones (CS) with 250-ms intervals. This paradigm tests climbing fiber-dependent conditional motor learning [41,42]. TcMAC21 mice showed impaired learning, failing to avoid obstacles following tone cues (Figure 1E, individual data points were graphed and summarized data displayed in an inset; 2way ANOVA repeated measures with Sidak’s Post-Hoc test, session5 post-rise *p*<0.0001, session5 pre-rise *p*=0.0351), indicating deficits in associative motor adaptation.

Cerebellar dysfunction often affects vocalization across neurological conditions [43]. Mouse models with cerebellar circuit mutations [44] and other DS preclinical models [45,46] consistently exhibit vocalization deficits, pointing to shared neural pathways underlying speech and motor control. Analysis of P8 pup isolation calls revealed that, although all mice produced separation calls during isolation (example spectrograph of these calls shown in Figure 1F), TcMAC21 pups made significantly more calls than their euploid siblings, with less frequency modulation (Figure 1G,H; Number of calls t_(22)_ = 2.347, *p*=0.0283, Tonality of USVs t_(22)_ = 3.078, *p*=0.0055, unpaired t-test), while call duration and mean frequency remained unchanged (Figure 1I,J; Mean call frequency t_(22)_ = 0.9520, *p*=0.3514, Call duration t_(22)_ = 1.497, *p*=0.1485, unpaired t-test). In maternal retrieval tests, increased USVs from TcMAC21 pups led to shorter retrieval latencies (Supplementary Figure 1G,H; 2way ANOVA mixed-effects, trisomy effect F_(1, 27)_ = 2.661, *p*=0.0045). However, TcMAC21 dams showed longer latencies retrieving euploid pups (Supplementary Figure 1I; 2way ANOVA mixed-effects, trisomy effect F_(1, 27)_ = 6.826, *p*=0.0145), indicating bidirectional disruption of social communication where TcMAC21 pups enhance vocalization while TcMAC21 dams show reduced maternal responsiveness.

### Trisomy cerebellar vermis sizes are disproportionately reduced and climbing fiber synapses are enlarged

**Figure 2:**
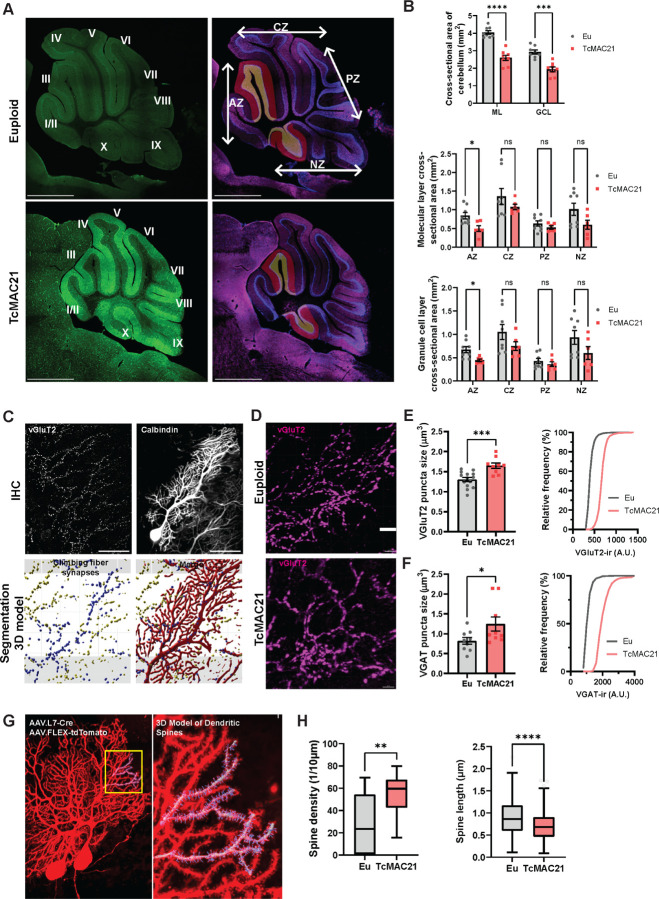
TcMAC21 cerebella feature disproportionally reduced anterior and nodular zones, altered pre- and postsynaptic morphology. **(A)**Representative euploid (top) and trisomy (bottom) midsagittal cerebellar sections. Fissures separate the vermis into lobes (left panels, designated by roman numerals). Anterior vermis (AZ) located anterior to the primary fissure, central vermis (CZ) located between the primary fissure and the horizontal fissure, and nodular vermis (NZ) included the nodulus, which is separated from the posterior vermis (PZ) by the posterolateral fissure (right panels). **(B)** The analysis of cerebellum morphology. The total cross- sectional area (top) of the molecular layer (ML) and granule cell layer (GCL). Data from the AZ, CZ, PZ, and NZ were discretely represented by (middle) the ML size or (bottom) the GCL size showed a significant decrease in AZ in TcMAC21 cerebella. **(C)** Confocal image showing an example of a Purkinje neuron transfected with AAV expressing tdTomato and double stained with vGluT2 (top panels); a high magnification view of a three-dimensional reconstruction of presynaptic vesicle clusters and dendritic shafts from the same neuron (bottom). Scale bar: 50mm. **(D)**Example of a stretch of synaptic vesicle clusters of climbing fiber synapses, labelled using VGluT2 antibody staining. Scale bar: 10 mm. Quantifications of **(E)** vGluT2 staining puncta size and intensity distribution, and **(F)** VGAT staining puncta size and intensity distribution. Both showed a significant trisomy effect of size enlargement. Bars represent mean ± S.E.M.. **(G)** A Confocal image showing an example of two Purkinje neurons transfected with AAV.L7- Cre/AAV.FLEX-tdTomato (red) (left), and a high magnification view of a dendritic segment from the neuron on the right with a three-dimensional reconstruction of spine heads and shafts (right). **(H)** Quantifications showing trisomy effects on an increased spine density and a decreased spine shaft length in TcMAC21 Purkinje neurons. Tukey boxplots represent median, the minimum and maximum values.

A previous study confirmed smaller cerebellar size in TcMAC21 mice [22], but our systematic assessment of TcMAC21 cerebellar lobules revealed disproportional hypoplasia in specific regions (Figure 2A,B; Molecular layer (ML) cross-section t_(14)_ = 7.821, *p*<0.0001, Granule cell layer (GCL) cross-section t_(14)_ = 5.569, *p*=0.0001, unpaired t-test). The anterior (IIII) and nodular lobes (IX/X) showed selective decreases in ML and GCL (Figure 2B; ML at anterior lobe (AZ) t_(12)_ = 2.935, *p*=0.0400, GCL at nodulus (NZ) t_(12)_ = 3.239, *p*=0.0386, unpaired t-test), indicating differential effects of HSA21 triplication across lobules.

Purkinje cells in the cerebellar cortex receive two types of excitatory inputs, the climbing fibers and the parallel fibers, and inhibitory inputs from stellate and basket cells in the molecular layer. We performed high-resolution confocal analysis of climbing fiber synapses and revealed enlarged VGluT2-positive terminals in TcMAC21 cerebella (Figure 2C) because there is a broad agreement that somatosensory feedback drives plasticity in these synapses, which are essential for cerebellar learning [47–51]. VGluT2-positive synapses extended to around 80% of molecular layer height (Supplementary Figure 2A-C). We observed an increased puncta size of trisomic VGluT2-positive synapses (Figure 2D,E; Puncta size t_(21)_ = 4.041, *p*=0.0006, unpaired t-test). VGluT2 immunoreactivity intensity was also increased, suggesting altered presynaptic vesicle content. Furthermore, analysis of inhibitory synaptic inputs to Purkinje cells showed increased inhibitory synapse size with decreased presynaptic vesicle pool in TcMAC21 mice (Figure 2F; Puncta size t(664) = 4.410, *p*<0.0001, unpaired t-test). While molecular layer interneurons coordinately provide negative feedback to control Purkinje neuron firing [52,53], there is also evidence for GABAergic deficits in individuals with DS [54,55]. These changes, combined with observed gait abnormalities [56], indicate disrupted climbing fiber-Purkinje cell connectivity [57] as a key pathogenic mechanism.

### Calcium homeostasis in developing Purkinje neuron regulates adult cerebellar afferent synapse

Next, we examined whether trisomy of HSA21 affects the spine morphology of Purkinje neurons. To sparsely label Purkinje neurons and their dendritic spines, we systemic delivered Purkinje neuron-specific minimal promoter (0.8-kb) [58] driven CRE virus (AAV.L7–6.Cre PHP.eB Serotype, 1 ×10^10^ VG per animal) and conditional tdTomato expression vector via intracisterna magna (ICM) delivery (Supplementary Figure 2D-F). We observed increased spine density but decreased spine length in TcMAC21 mice at P45 (Figure 2G,H; Spine density t_(31)_ = 3.455, *p*=0.0016, Spine length t(3427) = 16.80, *p*<0.0001, unpaired t-test), suggesting a shift toward immature, filopodia-like spines [59] less capable of supporting stable synaptic connections and plasticity.

One potential cause of the altered dendritic spine morphology is dysfunctional calcium buffering. Dendritic spines are the primary sites of synaptic input, and their size and shape are closely linked to synaptic strength and plasticity. Altered calcium buffering, resulting from impaired calcineurin activity, may lead to an increase in dendritic spine density, as seen in other models of synaptic dysfunction [60–62]. The overexpression of HSA21 genes like *DSCAM*, *RCAN1*, and *DYRK1A* in Down syndrome disrupts calcium homeostasis (Table 1), primarily by suppressing the calcineurin pathway [9,63]. This disruption results in elevated intracellular calcium levels due to impaired calcium buffering [64,65]. Given that calcium signaling is crucial for synaptic development, plasticity, and function [66,67], we hypothesize that this calcium dysregulation directly contributes to synaptic abnormalities in the cerebellar circuit. We expected that developmental perturbation of Purkinje neuron calcium homeostasis can have a variety of effects on cerebellar circuit formation thus creating phenotype resemblance to enlarged climbing fiber synapses in MAC21 (Figure 2D,E).

**Figure 3:**
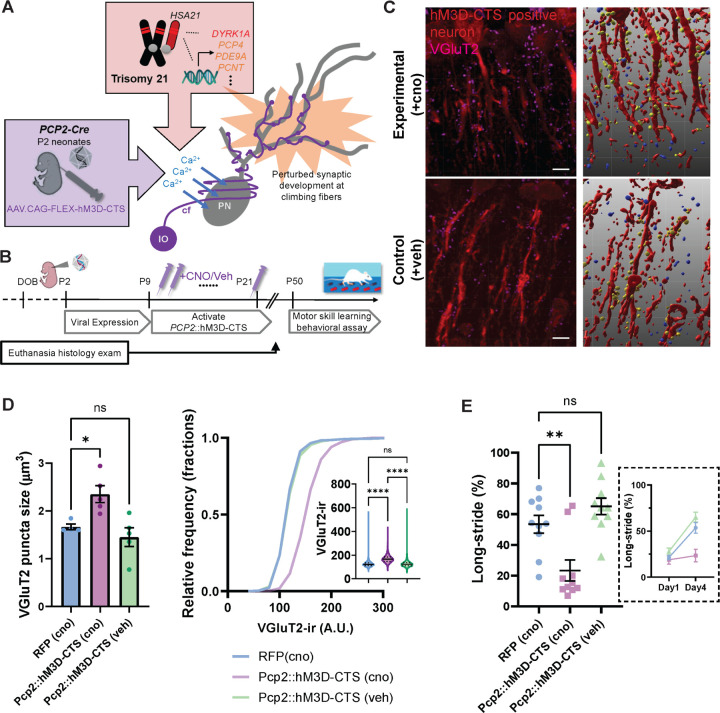
Developing Purkinje neuron calcium imbalance induces a persistent morphological change in cerebellar afferent synapses and a deficit in inter-limb adaptation task in adult. **(A)** Schematic to determine whether dysregulation of developing Purkinje neuron calcium balance leads to long-lasting changes in cerebellar circuit function. **(B)** Developing Purkinje neuron calcium dysregulation experimental timeline. CNO was administered from P9 to P21 to mice expressing hM3D-CTS or RFP in Purkinje neurons, and behavioral testing was conducted 30 days later at P50; an independent cohort was established for confocal analysis of climbing fiber synapses to control for cerebellar plasticity effects induced by motor training. **(C)** Representative confocal images (left) of Purkinje neurons express hM3D-CTS co-staining with VGluT2, and a three-dimensional reconstruction (right) of viral transduced dendrites (Red), neighboring presynaptic terminals (Yellow) and non-adjacent terminals (Blue), that were cno-treated (top) or vehicle-treated (bottom). Scale bar: 10 mm. **(D)** Quantification of VGluT2 staining puncta size and intensity showed a significant enlargement in climbing fiber afferent synapses in early postnatal period activated hM3D-CTS mice compared to controls. **(E)** CNO-treated hM3D-CTS mice showed a lower proportion of adapting a long-stride pattern. Individual data points of the last (Day4) training session were graphed and trend across days displayed in an inset. Data are presented as the mean ± S.E.M.; *p*(RFP vs. Pcp2::hM3D-CTS_veh_) = 0.3035, **p(RFP vs. Pcp2::hM3D-CTS_cno_) =0.0027; Ordinary ANOVA with post-hoc Dunnett’s test.

To test this hypothesis, we examined the developmental relationship between calcium buffering capacity and synaptic morphology in Purkinje neurons in vivo. To investigate whether these developmental defects are cell-autonomous to Purkinje neurons, we applied the Purkinje neuron-specific *Pcp2-Cre* to drive targeted recombination in these neurons beginning at postnatal day 2 (P2). We then used chemogenetic activation of primary cilia, a transient signaling organelle capable of controlling circuit formation [68], by coupling mutant GPCRs (DREADDs) with Gq to activate phospholipase C, leading to increased intracellular calcium upon clozapinen-oxide (cno) stimulation (Figure 3A). The injection contained either AAV.CAG.FLEX-hM3DCTS (*Pcp2::*hM3D-CTS group) or a control construct expressing dTomato (RFP group). We induced hM3D-CTS at P9–P21 with cno (1.0 mg/kg) or saline-only, given orally twice daily [69], during cerebellar circuit refinement (Figure 3B) [70,71]. We found increase intracellular calcium level in Purkinje neurons during postnatal developmental period has long lasting effect of enlarged climbing fiber synapses and increased VGluT2 immunoreactivity in their presynaptic terminals (Figure 3C,D; five biological replicates per treatment type; Puncta size analysis, 2way ANOVA with post-hoc Dunnett’s test *Pcp2::*hM3D-CTS (cno) vs. RFP (cno): adjusted *p*=0.0006, *Pcp2::*hM3D-CTS (cno) vs. *Pcp2::*hM3D-CTS (veh): adjusted *p*=0.0006; puncta intensity analysis, 2way-ANOVA with Dunnett’s post-hoc test *p*<0.0001). These findings suggest that the TcMAC21 mutants and manipulation of the intracellular calcium pathway converge on a common mechanism, leading to similar structural alterations at Purkinje cell synapses, indicative of a shared disruption in synaptic architecture and development.

Since the adult cerebellar afferent synapse phenotype bears similarity between TcMAC21 mice and Pcp2::hM3D-CTS (cno) mice, we next used inter-limb control adaptation behavior to determine the adult functional significance of perturbed intracellular calcium pathway in developing Purkinje neurons. We assessed locomotion performance in *Pcp2::*hM3D-CTS mice using ErasmusLadder and found training-induced step pattern change was suppressed in the cno-treated experimental hM3D-CTS expressing group when compared to control groups (vehicle-treated, or RFP expressing) (Figure 3E). Taken together, these results point to a longterm effect of the intracellular calcium disturbance neurons during neonate development on climbing fiber synapses to cerebellar Purkinje neurons, with an enlarged volume of the VGluT2 boutons accompanied by a decrease in locomotor performance.

### Trisomy of HSA21 alters the organization of Purkinje neuron sagittal stripe gene expression in developing and adult cerebellum

**Figure 4:**
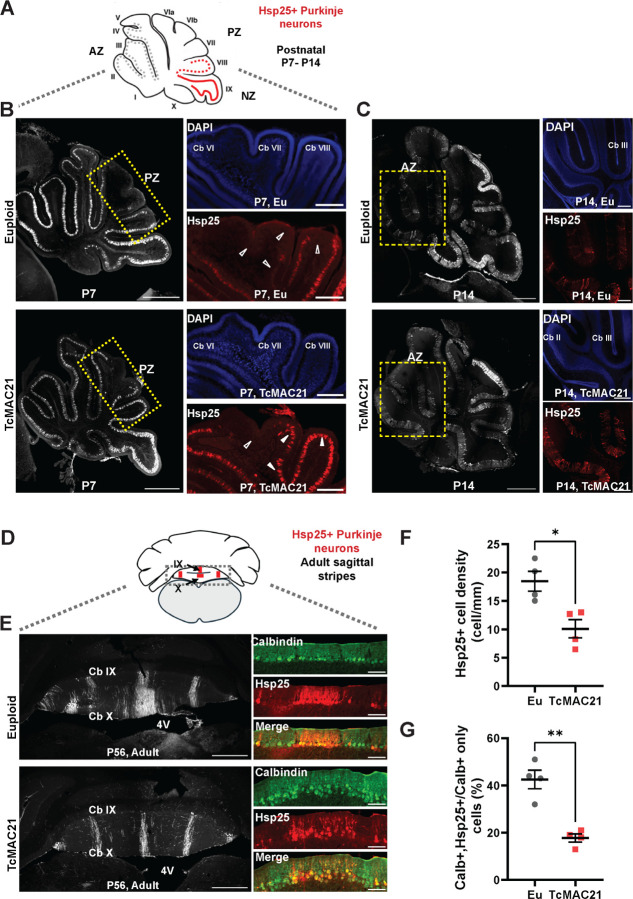
Developing and adult TcMAC21 cerebella exhibited disrupted Purkinje neuron subtype antigen Hsp25 expression pattern. **(A)** The sagittal schematics above the data panels indicate the lobules that show parasagittal stripes of Hsp25 (Red), or its absence (Gray); and correspond to the tissue sections shown in panels below. **(B)** At P7, Hsp25 immunoreactivity (ir) shows diminished expression in the posterior lobe (dotted rectangle) of euploid cerebella (*top*, empty arrowheads), while expression remains widespread in TcMAC21 mice (*bottom*, filled- arrowheads), indicating disrupted patterning. The presence of DAPI counterstaining indicated the absence Hsp25-ir was not due to artifact. Scale bars: 500 μm on left-panels and 100 μm on the right-panels **(C)** The atypical HSP25 distribution persists at P14 in TcMAC21 cerebella (*bottom*), differing from the typical restricted pattern seen in euploids (*top*). Note the expanded expression domain (dashed rectangle) compared to the normal restricted pattern. **(D)** In adult mice, the coronal schematics indicate Hsp25-positive Purkinje neurons show specific patterning in the nodular zone of control cerebella. **(E)** TcMAC21 mice display reduced Hsp25-positive Purkinje neurons in the medial parasagittal stripes, but wider parasagittal stripes were seen in euploid mice. Scale bars: 500 μm and 50 μm (insets). Quantification shows a significantly decrease in both the cell number **(F)** and percentage **(G)** of Hsp25+ Purkinje neurons in adult TcMAC21 mice.

Given that HSA21 showed regional changes to global cerebellar morphology and synaptic organization, we next investigated whether HSA21 also changed local cerebellar patterning. To determine whether TcMAC21 mice exhibit enhanced patterning abnormalities in the nodular zone of cerebellar vermis [72,73], we analyzed Purkinje neuron zonal patterning during early postnatal development and in adult. The majority of Purkinje neurons across the vermis and hemispheres express Hsp25 at the first week after birth [74], but the expression pattern in the anterior lobe is transient and diminishes over time. Indeed, we observed that Hsp25 immunoreactivity already was diminishing at the posterior lobe of the euploid cerebella, but that the expression remained widespread in TcMAC21 (Figure 4A,B), indicating disrupted patterning. This atypical organization persists into the second postnatal week (P14), potentially impeding circuit formation and refinement, contrary to the typical restricted distribution of Hsp25-positive (Hsp25+) Purkinje neurons (Figure 4C).

We then proceeded to examine whether there were differences in the adult zonal patterning. To determine the abundance of Purkinje neuron subtype, we measured the number of HSP25+ cells in the middle parasagittal zone in the lobules IX and X because of their consistent topographical patterning in adult (Figure 4D,E) [32,75]. In adult TcMAC21, we found that Hsp25+ cells were less abundant (Figure 4F; Cell number t_(6)_=3.491, *p*=0.0130, unpaired t test). To determine whether trisomy resulted in selective loss of the Hsp25+ cell type, we quantified the percentage of Purkinje neurons specification using Hsp25 co-labeled cells expressing the Purkinje neuronal marker Calbindin (Calb) in lobule IX. Reduced percentages of Hsp25+ neurons were reproducibly found in TcMAC21 mice (Figure 4G; Cell percentage t_(6)_=5.784 *p*= 0.0012, unpaired t test). These findings demonstrate that trisomy of HSA21 disrupts the typical zonal patterning of Purkinje neurons during early postnatal development, with abnormal persistence of Hsp25 expression in TcMAC21 mice, leading to altered cerebellar organization that may impede proper circuit formation and refinement into adulthood.

### Altered Cerebello-Thalamic Responses During Locomotion in TcMAC21 Mice

**Figure 5:**
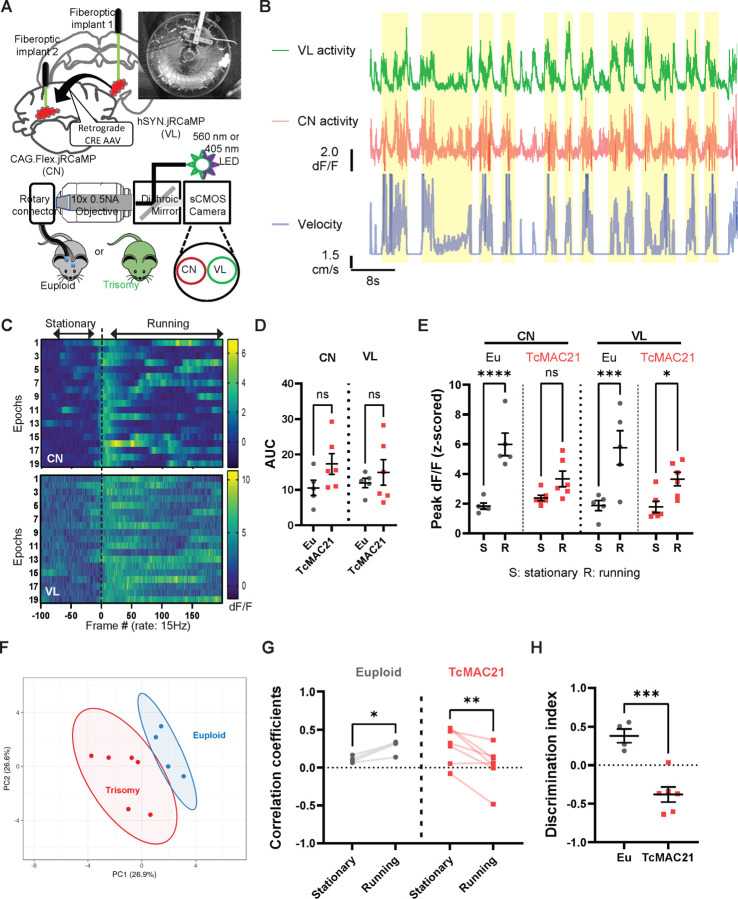
Trisomy21 alters the temporal pattern of the cerebellothalamic activation during locomotor activity. **(A)** Schematic setup. An optic bundle consist of two optical fibers that connect the optical cannula implanted at interposed nucleus (IPN) and ventrolateral nucleus of thalamus (VL) to the focal plane of objective. A dichroic mirror separates excitation and detection pathways, selects the detected wavelength range by emission filter. A complementary metal-oxide semiconductor (CMOS) camera sensor creates an image of the two-region fiber array. We used 560-nm LED to excite RCaMP. Upper right: example video camera image of voluntary wheel running with simultaneous two-region fiber photometry recording. **(B)** Example trial recordings. Top to bottom: bulk RCaMP ΔF/F (VL), ΔF/F (CN), and velocity traces recorded simultaneously across two regions from an euploid mouse. Shaded area: running epochs. **(C)** Example peristimulus time histogram (PSTH) responses from two brain regions of an euploid mouse. Top: CN recordings, bottom: VL recordings. Heatmap color in each row indicates dF/F amplitude of one running epoch. Dashed lines indicate onset running. **(D)** Average activity (area under Z scored responses) for euploid (n=4) vs TcMAC21 (n=6) during 10 min recordings. No significant differences, ANOVA followed by Sidak’s test. Data are mean ± SEM. **(E)**Locomotor states dependent changes in mean response magnitude (maximum Z scored response averaged across behavior epochs). Individual mice shown, including mean ± SEM. *p < 0.05, ***p < 0.001, ****p < 0.0001 by two-way RM-ANOVA with Sidak’s multiple comparisons. **(F)** Multidimensional analysis using the eight temporal and amplitude bulk activity (z-scored dF/F) measurements from interposed nucleus and motor thalamus of TcMAC21 (n=6) and euploid (n=4) mice during locomotor state transitions. Each data point represents one mouse, mapped in two principal component spaces. Neuronal responses during locomotor state transitions clustered according to genotype. **(G)** Pairwise Pearson’s Correlations of bulk activity between motor thalamus and cerebellar nuclei recordings during each locomotor epoch for euploid (n=4) and TcMAC21 (n=6) mice. Data points represent correlation mean across epochs per mouse, including mean ± SEM. Euploids show significant increased correlation, yet MAC21s show significant decreased correlation, during running phase. Significance found in CN-VL mean correlations: *p(Eu) =0.019, **p (TcMAC21) = 0.007 by Paired t test. **(H)**Discrimination Index (DI) for quantifying the change of neural synchrony between stationary versus running phases. DI was defined as the difference in mean Pairwise Pearson’s Correlations of bulk activity between motor thalamus and cerebellar nuclei recordings during each locomotor epoch, divided by the Sum of standard deviations (see METHOD). Data are presented as the mean ± S.E.M. ***p < 0.001 by Unpaired t test.

Given our observations of disrupted cerebellar parasagittal organization and altered climbing fiber innervation patterns in TcMAC21 mice, we investigated whether these developmental alterations impair error signal processing through cerebello-thalamic pathways during motor behavior. Three cerebellar nuclei (fastigial, interposed, and lateral) conduct feedback and coordination motor signals [76–79] through their projection to ventral thalamus to modulate thalamocortical networks [80,81]. Among these, we focused on the interposed nucleus, which we will refer to as cerebellar nuclei (CN) throughout this study. We reasoned that, by comparing the neural responses of CN and their downstream thalamic targets (ventrolateral nucleus of thalamus, VL) in TcMAC21 and euploid mice during voluntary locomotion, we can directly assess the impact of trisomy 21 on this critical motor feedback circuit. To simultaneously recording from both CN and VL, we employed multi-site in vivo fiber photometry recording [25]. Identification of the glutamatergic CN neurons projecting to motor thalamus was achieved with retrograde Cre virus (rgAAV-Cre) injection in the VL to tag projection neurons with conditional GECI (FLEX.jRCaMP1b) expression in the CN (Figure 5A). We implemented a free wheel-running task in head-fixed mice, allowing for precise tracking of movement states and velocity while simultaneously recording neural activity in the cerebellothalamic tract (CbT) using multi-site fiber photometry [25,82]. Due to the self-initiation nature of the task, we found variation between our mice in how motivated they were in engaging in locomotive activity; some engaged more in running bouts than others during a 25-minute testing session. Using “percentage of time engaging locomotives” as the measure of motivation, we found no motivational difference between Euploid and Trisomy mice (Supplementary Figure 1D-F).

We observed bulk neural responses in both CN and VL that were tuned to the onset of wheel running and were generally disengaged at the stationary phases (velocity=0) (Figure 5B,C; also see Supplementary Figure 3C-E). We quantified the area under curve (AUC) of dF/F trace and found no significant differences between euploid and TcMAC21 in average activity of any of the brain region (Figure 5D; ANOVA with Sidak’s Post-Hoc test, cerebellar nuclei p= 0.2461, ventrolateral thalamus p= 0.4676). However, during the wheel-running, we found that euploid mice displayed significantly higher magnitude of dF/F (z-scored) in both VL and CN compared to stationary phase (Figure 6E; Peak CN dF/F stationary vs. Running, Eu: *p*= 0.0037, TcMAC21: *p*= 0.0592; Peak VL dF/F stationary vs. Running, Eu: *p*= 0.0234, TcMAC21: *p*= 0.0109. Unpaired t test with Welch’s correction); but this locomotor state dependent activity distinction was absent in TcMAC21 mice. We extracted dynamical parameters of CN and VL activities during initiation or disengagement from running, including: latency to peak mean dF/F, 10% rise/fall, and peak correlation Pearson’s correlation of activity between simultaneously recorded neuronal traces, and analyzed these by principal component analysis (PCA) to reduce the dimensionality of the fiber photometry data. We found that the underlying neural dynamics captured by fiber photometry can segregate subjects based on genotype. The clusters of euploid and TcMAC21 mice had a centroid distance of 3.40 (Figure 5F; PC1: 30.1% variance, PC2: 23.4% variance).

To determine whether locomotor state modulates the neural synchrony between cerebellar nuclei and motor thalamus, we calculated stationary and running phases related to synchronicity. The increased CN-VL correlations (Pearson’s r during running epochs) were specific to euploid mice, while decreased CN-VL correlations were observed in TcMAC21 mice (Figure 6G; Pearson’s correlation coefficients, Eu: *p*=0.0197, TcMAC21: *p*=0.0076, Paired t test). To directly compare these between genotypes, we used a pattern discrimination index (DI) which measures how distinctly running and stationary states are reflected in inter-regional correlations (see METHOD). Euploid mice showed a DI of 0.3803, indicating robust statedependent modulation of neural synchrony. In contrast, trisomy mice exhibited a significantly lower DI of −0.3797 (Figure 5H; F_(5,3)_=1.804 *p* = 0.0007, Unpaired t test), suggesting impaired locomotor-dependent coordination between these regions.

## 4. DISCUSSION

We demonstrate that the TcMAC21 mouse model exhibits specific cerebellar-dependent motor and communication deficits that parallel human Down syndrome phenotypes. By employing the ErasmusLadder paradigm, which minimizes confounds from physical variables like hypotonia [83], we identified distinct impairments in inter-limb coordination and associative learning. These deficits mirror those seen in other cerebellar circuit mutations [23,56] and *Nlgn3*^KO^ autism models [57], suggesting shared mechanisms of cerebellar dysfunction across neurodevelopmental disorders.

Our findings reveal a potential developmental mechanism whereby trisomy 21 disrupts climbing fiber-Purkinje cell connectivity. The observed enlarged VGluT2 synapses and altered cerebellar nuclear output align with human imaging studies showing cerebellar afferent abnormalities in Down syndrome [84,85]. The vocal communication phenotype in TcMAC21 mice provides insight into the developmental origins of speech impairments in Down syndrome. The selective disruption of frequency modulation, rather than global vocalization deficits, suggests specific perturbations in circuits controlling vocal complexity [2,86]. The bidirectional impairment in pup-dam communication further indicates that trisomy 21 affects both expressive and receptive aspects of social communication, consistent with clinical observations. Altogether, these suggest that early perturbations in cerebellar circuit organization may underlie both motor and communication deficits.

Our analyses revealed two key alterations in TcMAC21 mice compared to euploid controls: elevated VL thalamic responses and delayed ramping of thalamic activity during locomotor initiation. The significantly higher VL activity in TcMAC21 mice, quantified by normalized area under the curve during running epochs, suggests aberrant thalamic activation during locomotion. This hyperactivity may reflect either compensatory mechanisms or maladaptive responses due to impaired cerebellar nuclear modulation of VL neurons. Additionally, the increased latency from locomotor onset to peak VL activity in TcMAC21 mice indicates impaired temporal processing of motor-related signals in the thalamus. The altered temporal precision in cerebellar nuclear output during locomotor state transitions likely reflects improper integration of climbing fiber error signals, potentially arising from disrupted Purkinje cell zonation patterns, altered climbing fiber synaptic properties, or both.

These physiological recordings uncovered reduced cerebello-thalamic synchrony during motor behavior, providing a circuit-level mechanism for impaired motor learning. The ability to recapitulate both synaptic and behavioral phenotypes through developmental manipulation of Purkinje cell calcium signaling establishes a causal link between early calcium dysregulation and persistent cerebellar dysfunction. Our findings suggest altered calcium homeostasis as a potential therapeutic target. However, rescue experiments require careful consideration, as previous work demonstrated that restoring cerebellar architecture through SAG treatment in Ts65Dn mice failed to improve vestibulo-ocular reflex adaptation despite normalizing granule cell numbers [87]. While inhibitory DREADDs could potentially normalize climbing fiber morphology by reducing Purkinje cell calcium influx, the complex interplay between granule cell development and Purkinje cell signaling suggests that combinatorial approaches targeting both pathways may be necessary for functional rescue - a hypothesis that warrants rigorous future investigation.

The cerebellum is consistently and significantly reduced in individuals with DS [3,4,88,89], aligning with their delayed motor development[90], poorer motor competence [34,36] and linguistic skills [91,92]. Surprisingly, Ts65Dn DS model did not show difference in accelerating rotarod test comparing to euploid mice [4,93]; other genetic models exhibited variable results [12,94]. Thus, a significant challenge in the field has been addressing underlying mechanisms of motor dysfunction in DS. Here, we demonstrated the TcMAC21 model offers opportunities to investigate how cerebellar circuit disruptions interact with other affected brain regions in Down syndrome. Future studies should examine the molecular pathways linking trisomy 21 to calcium dysregulation and explore therapeutic strategies targeting early cerebellar development.

## Supplementary Files

This is a list of supplementary files associated with this preprint. Click to download.

- MolPsySI.pdf

## Figures and Tables

**Table 1.** Gene triplication on human chromosome 21 controls intracellular calcium.

HSA21 gene	Effector	Mechanism of action	Reference
*DYRK1A*	GluN2A, CaMKIIδ, CEP97	Surface expression and channel activity of NMDA receptors, intracellular signaling molecules in the cytoplasm	PMID: 25368549 PMID: 26067684 PMID: 34787650
*PDE9a*	Phospholamban	elevating the cytoplasmic [Ca2+]	PMID: 28649129
*PCP4*	Calmodulin	mobilize [Ca2+] in context dependent manner	PMID: 10751438 PMID: 23204517
*PCNT*	Calmodulin,PCP2	Distribution of calcium-selective channel on primary cilia	PMID: 25031429 PMID: 15337773

**Table 2.** Details of statistical tests and results.

Figure Panel	Figure title	Statistical test	Sample size	Comparison
Fig. 1B	Missstep (session 1–4)	RM-ANOVA test; Sidak’s Post-Hoc test	Eu male: n = 9, MAC21 male: n = 9	Eu-MAC21, session1
				Eu-MAC21, session2
				Eu-MAC21, session3
				Eu-MAC21, session4
Fig. 1C	Steptime (session 1–4)	RM-ANOVA test; Sidak’s Post-Hoc test	Eu male: n = 9, MAC21 male: n = 9	Eu-MAC21, session1
				Eu-MAC21, session2
				Eu-MAC21, session3
				Eu-MAC21, session4
Fig. 1D	Percentage longstep (session 1–4)	2way ANOVA-RM	Eu male: n = 9, MAC21 male: n = 9	Eu-MAC21, session1
				Eu-MAC21, session2
Fig. 1E	Steptime (session 5–6)	2way ANOVA-RM; Sidak’s Post-Hoc test	Eu male: n = 9, MAC21 male: n = 9	Eu-MAC21, session5 post-rise
				Eu-MAC21, session6 post-rise
				Eu-MAC21, session5 pre-rise
				Eu-MAC21, session6 pre-rise
Fig. 1G	Call amount	Unpaired t-test	Eu pups: n = 12, MAC21 pups: n = 12	Eu vs, MAC21
	USV tonality	Unpaired t-test		Eu vs. MAC21
	USV frequency	Unpaired t-test		Eu vs. MAC21
	Call duration	Unpaired t-test		Eu vs. MAC21
Fig. 2B	Cross-sectional area, total cerebellum	Unpaired t-test	Eu: n = 8, MAC21: n = 8	Eu vs. MAC21, molecular layer
		Unpaired t-test		Eu vs. MAC21, Granule cell layer
	Cross-sectional area, molecular layer	Ordinary ANOVA; Sidak’s Post-Hoc test	Eu: n = 6, MAC21: n = 8	Eu-MAC21, Anterior Zone
				Eu-MAC21, Central Zone
				Eu-MAC21, Posterior Zone
				Eu-MAC21, Nadular Zone
	Cross-sectional area, granule cell layer	Ordinary ANOVA; Sidak’s Post-Hoc test	Eu: n = 6, MAC21: n = 8	Eu-MAC21, Anterior Zone
				Eu-MAC21, Central Zone
				Eu-MAC21, Posterior Zone
				Eu-MAC21, Nadular Zone
				
Fig. 2E	VGluT2 puncta size	Unpaired t test	Eu: n = 9, MAC21: n = 14	Eu vs. MAC21
	VGluT2-ir puncta intensity	Unpaired t test		Eu vs. MAC21
Fig. 2F	VGAT puncta size	Unpaired t test	Eu: n = 9, MAC21: n = 9	Eu vs. MAC21
	VGAT-ir puncta intensity	Unpaired t test		Eu vs. MAC21
Fig. 3D	VGluT2 puncta size	ANOVA test; Dunnett’s Post-Hoc test	Pcp2::hM3D-CTS+cno: n = 5, Pcp2::hM3D-CTS	RFP (cno) vs. Pcp2::hM3D-CTS (cno
	VGluT2-ir puncta intensity	ANOVA test; Tukey’s Post-Hoc test		RFP (cno) vs. Pcp2::hM3D-CTS (veh) RFP (cno) vs. Pcp2::hM3D CTS (cno) RFP (cno) vs. Pcp2::hM3D CTS (veh) Pcp2::hM3D-CTS (cno) vs. Pcp2::hM3D-CTS (veh)
Fig. 3E	Percentage longstep	RM-ANOVA test; Dunnett’s Post-Hoc test	Pcp2::hM3D-CTS+cno: n = 10, Pcp2::hM3D-CT	RFP (cno) vs. Pcp2::hM3D-CTS (cno) Day1
				RFP (cno) vs. Pcp2::hM3D-CTS (veh) Day1
				RFP (cno) vs. Pcp2::hM3D-CTS (cno) Day4
				RFP (cno) vs. Pcp2::hM3D-CTS (veh) Day4
Fig. 4F	Density of Hsp+ cells	Unpaired t test	Eu: n = 4, MAC21: n = 4	Eu vs. MAC21
Fig. 4G	Percentage of Hsp25+ cells	Unpaired t test	Eu: n = 4, MAC21: n = 4	Eu vs. MAC21
Fig. 5D	Area under curve at z-scored dF/F of CN	2way RM-ANOVA; Sidak’s Post-Hoc test	Eu: n = 5, MAC21: n = 6	Eu vs. MAC21
	Area under curve at z-scored dF/F of VL			
Fig. 5E	Peak dF/F (CN, z-scored), for euploid mice	2way RM-ANOVA; Sidak’s Post-Hoc test	Eu: n = 5, MAC21: n = 6	Stationary vs. Running
	Peak dF/F (CN, z-scored), for MAC21 mice		MAC21: n = 6	Stationary vs. Running
	Peak dF/F (VL, z-scored), for euploid mice		Eu: n = 5	Stationary vs. Running
	Peak dF/F (VL, z-scored), for MAC21 mice		MAC21: n = 6	Stationary vs. Running
Fig. 5G	Pearson’s correlation coefficients	Paired t test	Eu: n = 5	Stationary vs. Running
		Paired t test	MAC21: n = 6	Stationary vs. Running
Fig. 5H	Discrimination Index	Unpaired t test	Eu: n = 4, MAC21: n = 6	Eu vs. MAC21
